# Analysis of Outcomes After Endovascular Abdominal Aortic Aneurysm Repair in Patients With Abnormal Findings on the First Postoperative Computed Tomography Angiography

**DOI:** 10.1177/15266028211030539

**Published:** 2021-07-28

**Authors:** Anna C. M. Geraedts, Sana Mulay, Susan van Dieren, Mark J. W. Koelemay, Ron Balm

**Affiliations:** 1 Department of Surgery, Amsterdam University Medical Centres, Amsterdam Cardiovascular Sciences, Amsterdam, The Netherlands

**Keywords:** abdominal aortic aneurysm, endovascular aneurysm repair, postoperative complication, reintervention

## Abstract

**Purpose:**

Lifelong follow-up after endovascular abdominal aortic aneurysm repair (EVAR) is recommended due to a continued risk of complications, especially if the first postoperative imaging shows abnormal findings. We studied the long-term outcomes in patients with abnormalities on the first postoperative computed tomography angiography (CTA) following EVAR.

**Materials and Methods:**

This is a retrospective study of all consecutive patients who underwent elective EVAR for nonruptured abdominal aortic aneurysm (AAA) between January 2007 and January 2012 in 16 Dutch hospitals with follow-up until December 2018. Patients were included if the first postoperative CTA showed one of the following abnormal findings: endoleak type I–IV, endograft kinking, infection, or limb occlusion. AAA diameter, complications, and secondary interventions during follow-up were registered. Primary endpoint was overall survival, and other endpoints were secondary interventions and intervention-free survival. Kaplan-Meier analyses were used to estimate overall and intervention-free survival. Cox regression analyses were used to identify the association of independent determinants with survival and secondary interventions.

**Results:**

A total of 502 patients had abnormal findings on the first postoperative CTA after EVAR and had a median follow-up (interquartile range IQR) of 83.0 months (59.0). The estimated overall survival rate at 1, 5, and 10 years was 84.7%, 51.0%, and 30.8%, respectively. Age [hazard ratio (HR) 1.06, 95% confidence interval (CI) 1.05 to 1.10] and American Society of Anesthesiologists (ASA) classification (ASA IV HR 3.20, 95% CI 1.99 to 5.15) were significantly associated with all-cause mortality. Overall, 167 of the 502 patients (33.3%) underwent 238 secondary interventions in total. Fifty-eight patients (12%) underwent an intervention based on a finding on the first postoperative CTA. Overall survival was 38.4% for patients with secondary interventions and 44.5% for patients without (log rank; p=0.166). The intervention-free survival rate at 1, 5, and 10 years was 82.9%, 61.3%, and 45.6%, respectively.

**Conclusions:**

Patients with abnormalities on the first postoperative CTA after elective EVAR for infrarenal AAA cannot be discharged from regular imaging follow-up due to a high risk of secondary interventions. Patients who had a secondary intervention had similar overall survival as those without secondary interventions.

## Introduction

Endovascular aneurysm repair (EVAR) is widely used as the primary method of treating infrarenal abdominal aortic aneurysm (AAA).^1^ In the Netherlands, approximately 75% of elective operations for AAA are done with EVAR.^2,3^ The main downside of EVAR remains the risk of failure of endografts in the long run, with frequent need for secondary interventions. To assess prompt identification and treatment of graft-related complications, regular follow-up schemes are designed.^4–6^ International guidelines have given clear recommendations for post-EVAR surveillance.^7,8^ Currently, the European Society for Vascular Surgery (ESVS) guidelines recommend a computed tomography angiography (CTA) 30 days after EVAR. Patients must be evaluated for secondary intervention if this CTA detects an inadequate seal or an endoleak type I or III. If the first post-operative CTA shows adequate overlap and sealing zone, but an endoleak type II is present, sac expansion or shrinkage should be monitored.^7^ The current Society for Vascular Surgery (SVS) guidelines recommend a CTA in the first month after EVAR. Alarming findings, that is. endoleak type II, require surveillance at 6 months. If a new endoleak is detected close evaluation is necessary. If neither sac enlargement nor an endoleak is present yearly surveillance is suggested with either CTA or duplex ultrasound (DUS).^8^ The first postoperative CTA may therefore be an aid to decide on the required frequency of EVAR follow-up. Despite the recommendations from international guidelines, there is still reasonable doubt about the optimal regimen, the frequency of follow-up, and the imaging modality used for follow-up varies significantly between institutions.^9^ The appearance of abnormalities (<10 mm sealing zone and/or presence of any endoleak) on the first postoperative CTA has been shown to predict a high risk of complications after EVAR.^10,11^ Patients in the low-risk group had a 3.3% risk of AAA-related adverse events for up to 5 years following EVAR, vs 46.5% in the high-risk group.^10^

The current study was conducted to determine overall survival and the incidence of secondary interventions in patients with abnormal findings on the first postoperative CTA.

## Materials and Methods

### Study Design

This retrospective observational study was performed in accordance with the STROBE (Strengthening the Reporting of Observational Studies in Epidemiology) statement.^12^ The opt-out procedure was used to allow patients to object to participation within four weeks of notification, which is in accordance with the Dutch Code of Civil Procedure. The Medical Ethics Review Committee of the Amsterdam University Medical Centers, location Academic Medical Centre, Amsterdam, has confirmed that the Medical Research Involving Human Subjects Act (WMO) does not apply to our retrospective study. This study was conducted in accordance with the General Data Protection Regulation (AVG 2016) and the Medical Treatment Agreement Act (WGBO). The study was conducted in 16 Dutch medical centers with ample experience in EVAR.

### Patients

All consecutive patients with AAA who underwent elective EVAR in 16 medical centers between January 2007 and January 2012 were eligible for inclusion in this study. This provided a theoretical length of follow-up of 6 to 11 years by December 2018. Inclusion criteria were the presence of an infrarenal aortic or aortoiliac aneurysm treated with standard EVAR (no chimneys or fenestrations), and abnormal findings (endoleak type I–IV, endograft kinking, infection, or limb occlusion) on the first postoperative CTA, performed within 90 days after the initial operation. Patients with a non-ruptured, symptomatic AAA were also included. Patients with ruptured or isolated iliac aneurysms or patients with previous abdominal aortic surgery were excluded.

### Data Collection

Data were collected from patient medical records by 2 investigators (AG, SM) and entered into a secured study-specific database. Data included baseline characteristics and anatomical parameters on the preoperative and first postoperative CTA. Anatomical parameters were obtained from radiology reports. The baseline characteristics collected were gender, age at time of surgery, American Society of Anesthesiologists (ASA) classification, and endograft type. Anatomical parameters were AAA diameter (anterior-posterior), neck length, and the maximum diameters of the iliac arteries. Follow-up data included all surveillance imaging studies [DUS, CTA, magnetic resonance angiography (MRA)], including the AAA diameter, complications, interventions, and mortality. Mortality data were ascertained by record linkage between the study population and the national death register.

### Definitions

All imaging studies were assessed or supervised by local experienced radiologists and the data from these reports were extracted for the current study. We were not able to review all individual imaging studies within the context of this study. Abnormal radiological findings after the initial postoperative CTA included: endoleak types I, II, III, and IV, endograft migration, kinking or infection, limb occlusion and other. Endoleaks were defined as follows: type I—leaks from proximal (Ia) and/or distal (Ib) sealing zones, type II—secondary to patency of aortic branches, type III—separation of graft components, and type IV—flow from porous fabric. Graft migration was defined as an absolute change of ≥10 mm in relation to anatomical landmarks.^13^ Limb occlusion was defined as outflow obstruction within the limb of the endograft. Limb kinking, sometimes in combination with limb occlusion, was diagnosed during follow-up or identified if patients became symptomatic with lower extremity ischemia.^13^ Endograft infection was defined as peri-graft fluid or gas detected on CTA.^14^ Patients were divided into 3 groups according to the observed AAA diameter over time, that is, increased diameter, stable diameter, and decreased diameter. A 5-mm threshold was selected for diameter changes in the aneurysm sac between the follow-up imaging studies or a 5-mm difference from the initial postoperative AAA diameter.^13^ Sac growth was defined as an increase of >5 mm between 2 consecutive imaging studies or if there was >5-mm growth on comparison with the initial postoperative AAA diameter. AAA sac shrinkage was defined as a decrease of >5 mm between 2 consecutive imaging studies or if there was >5-mm shrinkage on comparison with the initial postoperative AAA diameter. An AAA was considered stable if no sac growth or shrinkage occurred.

### Surveillance Programs

Each center provided their respective surveillance protocol (Supplemental Table 1). Surveillance protocols varied by center, though CTA and DUS were the 2 dominant modalities used for surveillance in the first year. After the first year, most patients were assessed with DUS.

### Endpoints

The primary endpoint was overall survival. Secondary endpoints included intervention-free survival, type, frequency, and indication for secondary interventions during follow-up and aneurysm rupture. Abnormal findings on the first postoperative CTA and during follow-up were described in detail.

### Statistical Analysis

The Shapiro-Wilk test, box plots, and histograms were used to assess if continuous data followed the normal distribution. Normally distributed continuous variables are presented as mean ± standard deviation (mean ± SD), otherwise as median with interquartile range (IQR). Differences between groups were assessed using the Student’s *t* test, Kruskal-Wallis *H* test, or Mann-Whitney *U* test, as appropriate. Categorical variables were presented as numbers and percentages, and differences between groups were assessed using the Pearson χ^2^ or Fisher’s exact 2-tailed test, as appropriate. Intervention-free survival and secondary interventions were censored at the last contact with the hospital for imaging. Mortality data from the national death register were used to calculate overall survival and censored at time of death or December 2018. Kaplan-Meier survival analyses were used to estimate overall survival and intervention-free survival. Differences in survival between groups were assessed with the log-rank test. Univariable and multivariable Cox regression analyses were used to determine the hazard ratios (HRs) and 95% confidence intervals (CIs) of several variables associated with mortality and secondary interventions. Age, gender, AAA diameter, change in aneurysm diameter, ASA classification, endograft type, neck length, and maximum iliac diameter were included in univariable Cox regression. Variables with p-values less than 0.2 in univariable Cox regression were entered into a multivariable Cox regression model to identify independent determinants associated with mortality and secondary intervention (backward selection). In addition to account for missing values, sensitivity analysis was performed using imputation (10 sets) based on predictive mean matching. A 2-sided p-value <0.05 was considered to be statistically significant. All statistical analyses were performed with SPSS software 26 (IBM Corp, Armonk, NY, USA).

## Results

Between January 2007 and January 2012, 1734 of 2279 patients who underwent elective EVAR had normal findings on the first CTA. A total of 43 patients were excluded from this study since no postoperative CTA was performed within 90 days. In total, 502 (22%) patients (49 women) with abnormal findings on the first postoperative CTA were included in the study, 52 of whom underwent EVAR for a non-ruptured symptomatic AAA. The mean (SD) age was 74.4 years (7.5) at the time of EVAR. The mean (SD) preoperative AAA diameter was 6.4 cm (1.2). Baseline characteristics are presented in Table 1. The median (IQR) follow-up until the last imaging study was 52.4 months (59.3), and the median follow-up until death or December 2018 was 83.0 months (59.0).

**Table 1.** Baseline Characteristics.

Variable	Missing	Overall Group (N=502)
Age, y, mean (SD)	0	74.4 (7.5)
Sex, male, n (%)	0	433 (86.3)
AAA diameter, cm, mean (SD)	0	6.4 (1.2)
Neck length, cm, mean (SD)	157	2.9 (1.2)
ASA classification, n (%)	0	
I		4 (0.8)
II		194 (38.6)
III		279 (55.6)
IV		25 (5)
Endograft, n (%)	0	
Endurant (Medtronic)		166 (33.1)
Talent (Medtronic)		31 (6.2)
Excluder (Gore)		97 (19.3)
Zenith (Cook)		189 (37.6)
Powerlink (Endologix)		7 (1.4)
Anaconda (Vascutek)		3 (0.6)
Other		9 (1.8)
Maximum iliac diameter, cm, mean (SD)	185	1.6 (0.7)

Abbreviations: AAA; abdominal aortic aneurysm, ASA; American Society of Anesthesiologists.

### Overall Survival

**Figure 1.**
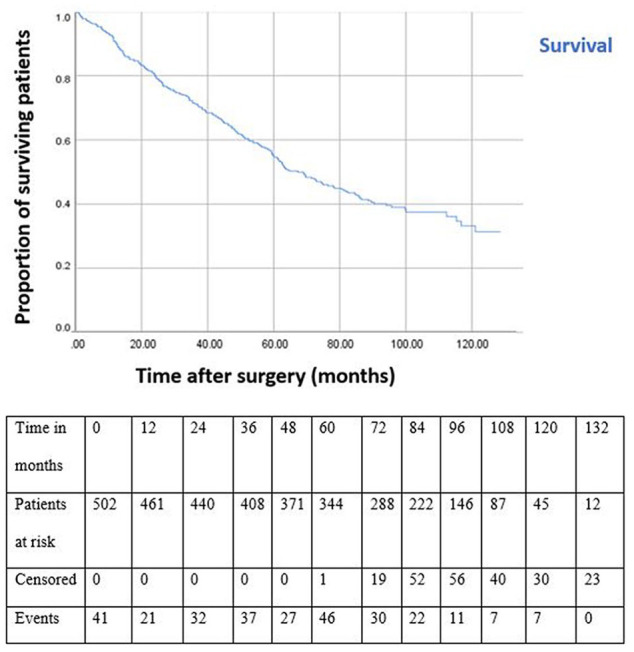
Kaplan-Meier overall survival curve for patients treated via endovascular aneurysm repair. All standard errors at each time point are less than 10%.

We identified 280 deaths among the 502 EVAR patients. Ninety-nine of 280 patients (35.4%) died of non-aneurysm-related causes, and 26 patients (9.3%) died of AAA-related causes [9 rupture, 11 after secondary intervention, 2 perioperative, 4 EVAR-related delayed deaths (eg, end-stage renal disease)]. The median (IQR) time to death for aneurysm-related causes was 34.18 months (59.21). In 155 cases, the cause of death was unknown (55.4%). Overall survival was censored at death or December 2018 and was 84.7%, 66.5%, 51.0%, and 30.8% at 1, 3, 5, and 10 years, respectively (Figure 1). Overall survival at 10 years was 38.4% for patients with secondary interventions and 44.5% for patients without (log rank; p=0.166). No difference was found in overall survival if patients underwent 1, 2, 3, or more secondary interventions (p=0.106). A total of 88 deaths (52.7%) occurred in the group with secondary interventions and 192 (57.5%) in the group with no interventions (p=0.309). There was no significant difference in overall survival for patients who underwent EVAR for an asymptomatic (58.1%) or symptomatic (65.3%) AAA, p=0.145. Overall 30-day mortality following the primary EVAR procedure for the entire cohort was 0.6% (3/502). Age, ASA classification IV, and type of endograft were significantly associated with mortality by univariable Cox regression analysis. In multivariable Cox regression analysis, age and ASA classification IV remained significantly associated with mortality (Table 2). In sensitivity analysis type of endograft became significantly associated with mortality (Supplemental Table 2).

**Table 2.** Cox Hazard Univariable and Multivariable Analysis of the Effect of Different Variables on Mortality and Secondary Interventions.

	Univariable Analysis				Multivariable Analysis			
	Mortality		Secondary Interventions		Mortality		Secondary Interventions	
	Hazard Ratio	95% CI	Hazard Ratio	95% CI	Hazard Ratio	95% CI	Hazard Ratio	95% CI
Age^a^	1.060	1.041 to 1.079	0.987	0.968 to 1.007	1.064	1.045 to 1.083		
Gender	0.956	0.690 to 1.322	0.783	0.522 to 1.175				
ASA I/II	Reference	Reference	Reference	Reference	Reference	Reference		
ASA III	1.070	0.836 to 1.371	1.187	0.859 to 1.641	1.100	0.854 to 1.416		
ASA IV^a^	2.899	1.814 to 4.633	3.166	1.765 to 5.678	3.203	1.994 to 5.145		
Neck length^b^	1.016	0.903 to 1.144	0.815	0.689 to 0.964			0.791	0.661 to 0.947
Endurant (Medtronic)	Reference	Reference	Reference	Reference				
Talent (Medtronic)	0.640	0.382 to 1.072	0.642	0.306 to 1.346				
Excluder (Gore)	0.706	0.505 to 0.986	0.733	0.467 to 1.150				
Zenith (Cook)	0.707	0.537 to 0.930	0.959	0.674 to 1.365				
Other	0.413	0.181 to 0.939	0.953	0.435 to 2.087				
AAA diameter^b^	1.048	0.949 to 1.159	1.111	0.979 to 1.262			1.223	1.045 to 1.432
Maximum iliac diameter	1.103	0.925 to 1.315	1.279	1.036 to 1.578				
Change in AAA diameter								
Stable/decrease	Reference	Reference	Reference	Reference			Reference	Reference
Increase^b^	1.013	0.756 to 1.357	2.335	1.687 to 3.232			2.262	1.546 to 3.308

Abbreviations: AAA; abdominal aortic aneurysm, ASA; American Society of Anesthesiologists.

a Variables significantly related to mortality in multivariable analysis.

b Variables significantly related to secondary interventions in multivariable analysis.

### Intervention-Free Survival

**Figure 2.**
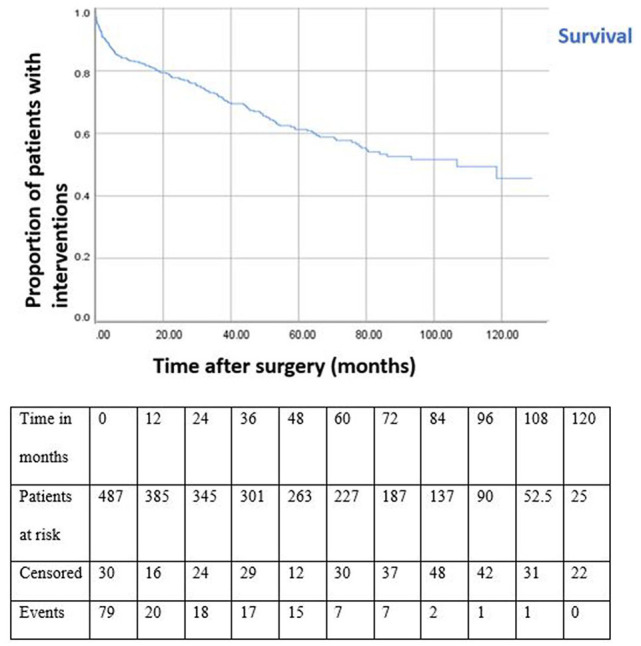
Kaplan-Meier intervention-free survival curve for patients treated with endovascular aneurysm repair. All standard errors at each time point are less than 10%.

The intervention-free survival at 1, 3, 5, and 10 years was 82.9%, 72.4%, 61.3%, and 45.6%, respectively (Figure 2). Univariable Cox regression analysis showed ASA classification, type of endograft, AAA diameter, maximum iliac diameter, and change in aneurysm diameter to be significantly related to a secondary intervention. In multivariable Cox regression analysis, neck length, AAA diameter, and increase in AAA diameter were significantly associated with secondary interventions (Table 2). After imputation ASA classification IV, neck length, type of endograft, and increase in AAA diameter remained significantly related to secondary interventions (Supplemental Table 2). In all, 155 of 238 secondary interventions (65.1%) were managed by endovascular techniques, and 41 (17.2%) required open surgical repair. A total of 43 (18.1%) comprised other types of intervention such as diagnostic angiograms or stent placement in the renal artery.

### Secondary Interventions

A total of 238 secondary interventions were performed in 167 patients; 167 patients needed 1 intervention, 43 patients also needed a second intervention, 18 patients a third intervention, 6 patients a fourth intervention, 2 patients a fifth and sixth intervention, and 1 patient needed 7 interventions in total. The most common indications for secondary intervention included type II endoleak, limb occlusion, and type I endoleak (Table 3). The most common adjuncts required were coil embolization of collateral channels for type II endoleak and extension involving deployment of secondary endoluminal prosthesis within the primary prosthesis, mainly for type I endoleak. No difference was observed in overall survival between patients with a type II endoleak who underwent a secondary intervention for a type II endoleak and those who did not, p=0.750. The 30-day mortality after a secondary intervention was 5.4% (9/167) and patients died due to aneurysm rupture, endograft infection or conversion to open surgery. Secondary interventions occurred after a median (IQR) of 30.0 months (57.9) after primary EVAR. Patients with greater iliac diameters did not have higher secondary intervention rates, p=0.204.

**Table 3.** Indication and Management for Intervention After Endovascular Aneurysm Repair.

Indication	Management	Number
Type I endoleak (n=54)	Balloon dilatation	3
	Additional mesh stent	7
	Aortic cuffs	10
	Extension	15
	Coil embolization	7
	Conversion	4
	Other	8
Type II endoleak (n=60)	Aortic cuffs	9
	Aortic cuffs + extension	2
	Coil embolization	47
	Conversion	2
Type III endoleak (n=10)	Balloon dilatation	2
	Additional mesh stent	1
	Aortic cuffs	2
	Extension	2
	Coil embolization	1
	Conversion	1
	Other	1
Endograft kinking (n=22)	Thrombectomy/embolectomy/endarterectomy	1
	Bypass graft	1
	Balloon dilatation	1
	Additional mesh stent	4
	Aortic cuffs	3
	Extension	5
	Conversion	3
	Other	4
Endograft migration (n=2)	Extension	1
	Conversion	1
Limb occlusion (n=55)	Trombectomy/embolectomy/endarterectomy	11
	Bypass graft	6
	Balloon dilatation	6
	Additional mesh stent	5
	Extension	4
	Hand sewn anastomosis	1
	Conversion	5
	Other	17
Endograft infection (n=5)	Conversion	1
	Other	4
Other (n=30)	Thrombectomy/embolectomy/endarterectomy	5
	Bypass graft	2
	Balloon dilatation	1
	Additional mesh stent	3
	Aortic cuffs	1
	Extension	7
	Coil embolization	1
	Conversion	2
	Other	8

### Asymptomatic and Symptomatic Presentation

Fifty-eight patients (12.0%) underwent an intervention as a consequence of a finding seen on the initial postoperative CTA (Table 4). Another 99 patients underwent a secondary intervention guided solely by surveillance imaging during follow-up. The most common abnormalities were endoleaks type I and II. An additional patients presented with symptoms that required a secondary intervention, independent of routine surveillance imaging: 2 patients became symptomatic due to lower limb ischemia, 5 patients presented with symptoms, and this appeared to be a rupture due to a type I endoleak and 1 patient’s aneurysm ruptured due to a type II endoleak. In total, 14 of 502 patients (2.8%) presented with AAA rupture. Four patients underwent endovascular repair after rupture, 3 patients open repair, and in 7 patients palliative care was provided. The median time to rupture was 53.1 months (range 1.38–121 months). Eleven patients (78.6%) died after rupture.

**Table 4.** Radiological Findings From the Initial Postoperative Computed Tomography Angiography and Following Interventions Within 90 Days.

Radiological Finding	n (%)	Intervention
Endoleak type I	49 (10)	12
Endoleak type I + II	9 (2)	7
Endoleak type I + III	1 (0.2)	1
Endoleak type I + kinking	2 (0.4)	1
Endoleak type I + other	6 (1.2)	
Endoleak type II	302 (60)	
Endoleak type II + III	7 (1.4)	1
Endoleak type II + kinking	1 (0.2)	
Endoleak type II + limb occlusion	2 (0.4)	
Endoleak type II + other	14 (2.8)	
Endoleak type III	15 (3)	1
Endoleak type III + kinking	2 (0.4)	1
Endograft kinking	27 (5.4)	3
Endograft kinking + limb occlusion	3 (0.6)	2
Endograft kinking + other	2 (0.4)	
Limb occlusion	26 (5.2)	18
Limb occlusion + other	2 (0.4)	
Endograft infection	3 (0.6)	1
Other	29 (5.8)	9
Total	502 (100)	58

### Change in Aneurysm Sac Size

During follow-up, in 317 patients the AAA diameter decreased or remained stable over time. A total of 109 patients had an increase in AAA diameter. Fifty-three patients attended fewer than two follow-up visits, and in 23 patients the AAA diameter fluctuated thus these measurements were not considered reliable and therefore excluded from further analysis. Patients with an increase in AAA diameter underwent significantly more secondary interventions than those with a stable or decreased AAA diameter (60.2% vs 26.7%; p<0.001). The estimated rate of freedom from secondary interventions 5 years after EVAR was 74.9% and 43.0% for patients with a decreased or stable versus increased AAA diameter, respectively (log rank; p<0.001; Supplemental Figure 1). The estimated overall survival rate 5 years after EVAR was 71.4% and 75.0% for patients with a decreased or stable versus an increased AAA diameter, respectively (log rank; p=0.931; Supplemental Figure 2).

## Discussion

The possibility to select patients with a particularly high incidence of complications and secondary interventions following EVAR, who might require a more vigilant follow-up regimen, will benefit patients and physicians. Both international guidelines recommend strict imaging surveillance enabling elective intervention of potentially fatal complications that can suddenly occur, like rupture or occlusion.^7,8^ However, we found similar overall 10-year estimated survival rates in patients with (38.4%) and without (44.5%) secondary interventions. One-third of patients in the present cohort underwent a secondary intervention during follow-up, the majority by means of an endovascular technique. Results derived from this study can inform patients and vascular surgeons regarding the probability of undergoing secondary interventions.

This study confirms the results of a single-center study where no association was found between survival and secondary interventions at 5 years (p=0.21).^15^ Current literature is ambiguous regarding the beneficial effect of secondary interventions on survival. Recently published data from our research group compared patients with and without a type II endoleak, which showed that overall survival was unaffected by the presence of a type II endoleak (p=0.537).^16^ However, this type II endoleak study included patients with and without abnormalities at their initial CTA, therefore, the results are not directly comparable. Baderkhan et al^11^ also demonstrated no difference in overall survival in patients classified as high (sealing zone less than 10 mm and/or presence of an endoleak) and low risk on the first postoperative CTA of adverse events after EVAR (log rank; p=0.077). This is in contrast with preceding large multicenter studies in which aneurysm related mortality was higher in patients who underwent (major) secondary interventions.^17,18^ This can partly be declared by the difference in outcome, since our primary outcome was all-cause mortality and not aneurysm-related mortality. After imputation, type of endograft was significantly associated with all-cause mortality. We do not have an explanation for this observation which might be due to other comorbidities that were not recorded in our database (residual confounding).

The intervention-free survival rate for patients with abnormal findings at the first postoperative CTA at 5 and 10 years was 61.3% and 45.6%, respectively. This is similar to the 5-year estimate for freedom from aneurysm-related adverse events of 52% in high-risk patients.^10^ In our cohort, multivariable Cox regression analysis found an association between neck length, increase in AAA diameter, and a large initial aneurysm diameter with a high secondary intervention rate as reported in previous literature.^19–21^ Almost 12% of patients underwent a secondary intervention within the first three months after EVAR. This is high in comparison with data from preceding reports,^4,22,23^ but could be explained by the fact that we only included patients with abnormalities at their initial postoperative CTA. After this period the need for secondary interventions continues to remain stable over time similar to other long-term studies.^24,25^

The majority of patients with an abnormal CTA that underwent a secondary intervention were asymptomatic and underwent their first intervention due to surveillance imaging after EVAR. A smaller proportion presented with symptoms, all of whom needed secondary intervention. Previous studies have reported on secondary interventions after EVAR solely initiated by symptoms instead of imaging surveillance.^26,27^ These findings are difficult to compare with our results since we specifically focus on the high-risk group of patients.

This study is limited by its retrospective and observational nature. Only patients who underwent EVAR up to January 2012 have been included, while over recent years devices have been improved, and vascular surgeons have become more experienced in EVAR. However, retrospective analysis is necessary to study long-term study outcomes. We obtained data from radiology reports and therefore we could not identify how many of the primary procedures were performed according to the instructions for use (information bias). Nevertheless, our study reflects real-world data from a large nationally representative sample of patients. The median follow-up to the date of the last hospital visit was 31 months shorter than follow-up with regard to survival status. This is in part explained by the fact that clinical follow-up of patients was transferred back to referring hospitals the patient attended prior to the intervention. As we did not have information on complications or secondary interventions after handing these patients over, the total event rate may be underestimated and the intervention-free survival overestimated. With regard to the imputed variables, 3 differences were observed. After imputation, type of endograft was significantly associated with all-cause mortality and secondary interventions, regarding the outcome secondary interventions ASA IV became associated but the initial AAA diameter was not significantly associated anymore. These findings may be coincidental as we have no explanation for this. The lack of knowledge of causes of death is also a limitation of this study. Postmortem examination in the Netherlands is only performed on indication after trauma and on selected patients who die in the hospital, and therefore it was not possible to determine the proportion of aneurysm-related deaths. The 2.8% of patients presented with rupture after EVAR could be an underestimation. The strengths of this study lie in the long-term follow-up of EVAR patients with abnormalities on first postoperative imaging, and in the large number of patients which gives an accurate real-world reflection of EVAR patients in the Netherlands.

## Conclusions

The current study revealed that if patients have abnormalities at their initial CTA there is a high risk of undergoing secondary intervention during follow up and therefore these patients cannot be discharged from regular imaging surveillance following EVAR. Additionally, clinical (age, ASA classification) and anatomical parameters (neck length, large initial AAA diameter, increase in AAA diameter) are revealed that may provide an increased risk of secondary interventions and all-cause mortality after EVAR requiring further consideration both in research and clinical fields.

## Supplemental Material

sj-pdf-1-jet-10.1177_15266028211030539 – Supplemental material for Analysis of Outcomes After Endovascular Abdominal Aortic Aneurysm Repair in Patients With Abnormal Findings on the First Postoperative Computed Tomography Angiography Click here for additional data file. Supplemental material, sj-pdf-1-jet-10.1177_15266028211030539 for Analysis of Outcomes After Endovascular Abdominal Aortic Aneurysm Repair in Patients With Abnormal Findings on the First Postoperative Computed Tomography Angiography by Anna C. M. Geraedts, Sana Mulay, Susan van Dieren, Mark J. W. Koelemay and Ron Balm in Journal of Endovascular Therapy

sj-pdf-2-jet-10.1177_15266028211030539 – Supplemental material for Analysis of Outcomes After Endovascular Abdominal Aortic Aneurysm Repair in Patients With Abnormal Findings on the First Postoperative Computed Tomography Angiography Click here for additional data file. Supplemental material, sj-pdf-2-jet-10.1177_15266028211030539 for Analysis of Outcomes After Endovascular Abdominal Aortic Aneurysm Repair in Patients With Abnormal Findings on the First Postoperative Computed Tomography Angiography by Anna C. M. Geraedts, Sana Mulay, Susan van Dieren, Mark J. W. Koelemay and Ron Balm in Journal of Endovascular Therapy

sj-pdf-3-jet-10.1177_15266028211030539 – Supplemental material for Analysis of Outcomes After Endovascular Abdominal Aortic Aneurysm Repair in Patients With Abnormal Findings on the First Postoperative Computed Tomography Angiography Click here for additional data file. Supplemental material, sj-pdf-3-jet-10.1177_15266028211030539 for Analysis of Outcomes After Endovascular Abdominal Aortic Aneurysm Repair in Patients With Abnormal Findings on the First Postoperative Computed Tomography Angiography by Anna C. M. Geraedts, Sana Mulay, Susan van Dieren, Mark J. W. Koelemay and Ron Balm in Journal of Endovascular Therapy
