# Supplementary material for: Analysis of Outcomes After Endovascular Abdominal Aortic Aneurysm Repair in Patients With Abnormal Findings on the First Postoperative Computed Tomography Angiography
Source: J Endovasc Ther. 2021 Jul 28;28(6):878–87. doi: 10.1177/15266028211030539 (PMC8573614; doi:10.1177/15266028211030539)
Supplement: sj-pdf-1-jet-10.1177_15266028211030539 – Supplemental material for Analysis of Outcomes After Endovascular Abdominal Aortic Aneurysm Repair in Patients With Abnormal Findings on the First Postoperative Computed Tomography Angiography [file sj-pdf-1-jet-10.1177_15266028211030539.pdf]

Supplemental Figure legends

Supplemental Figure 1: Kaplan-Meier for freedom from secondary intervention according to change in abdominal aortic aneurysm diameter (Log rank; $P < .001$). All standard errors at each time point are below 10%.

Supplemental Figure 2: Kaplan-Meier for overall survival according to change in abdominal aortic aneurysm diameter (Log rank; $P < .931$). All standard errors at each time point are below 10%.
