# Supplementary material for: Analysis of Outcomes After Endovascular Abdominal Aortic Aneurysm Repair in Patients With Abnormal Findings on the First Postoperative Computed Tomography Angiography
Source: J Endovasc Ther. 2021 Jul 28;28(6):878–87. doi: 10.1177/15266028211030539 (PMC8573614; doi:10.1177/15266028211030539)
Supplement: sj-pdf-2-jet-10.1177_15266028211030539 – Supplemental material for Analysis of Outcomes After Endovascular Abdominal Aortic Aneurysm Repair in Patients With Abnormal Findings on the First Postoperative Computed Tomography Angiography [file sj-pdf-2-jet-10.1177_15266028211030539.pdf]

Supplemental Table 1: Surveillance protocols in participating medical centers

**Centre**	**First imaging**	**Yearly thereafter**
1	A CT angiogram (CTA) after 4-6 weeks and clinical visit. No abnormalities: yearly Duplex ultrasound (DUS). Abnormalities: CTA is repeated. Endoleak type I/III: intervention.	DUS
2	Two-week clinical visit. A CTA, plain film x-ray and clinical review at 3 months post-procedure. CTA at 12 months. DUS + plain film x-ray at years 2,3 and 5. CTA at 5-years post-procedure.	DUS, 1/5 years CTA
3	6 weeks CTA. No abnormalities: DUS after 6 months and thereafter yearly. Type I endoleak: intervention or strict surveillance, type II: normal follow-up.	DUS, CTA if new endoleak and/or sac size increase
4	First day after discharge consultation via telephone. Clinical visit after two weeks. Six weeks clinical review + CTA.	DUS
5	CTA after 4 weeks.	DUS + Ankle-Brachial index (ABI) + biplanar plain film x-ray. CTA if new endoleak and/or sac size increase

6	Clinical visit + DUS 6-8 weeks. DUS after 3 months.	DUS + plain film x-ray every 2 years
7	CTA at 6 weeks, color-flow DUS at 6 months	Color-flow DUS<br><br>On indication: CTA
8	CTA at 4 weeks and 12 months	DUS
9	CTA at 4 weeks and 12 months	DUS
10	CTA at 4 weeks and 12 months	DUS, 1/5 years CTA
11	6-week clinical visit + CTA.	DUS or CTA every 5 years
12	CTA at 6 weeks, CTA or DUS + plain film x-ray at 6 months	DUS + plain film x-ray
13	6-week clinical visit + DUS, DUS at 3 months	DUS
14	CTA at 4 weeks and 12 months. No abnormalities: yearly DUS. Abnormalities: CTA at 3 months, endoleak I/III: intervention.	DUS
15	CTA at 6 weeks + biplanar plain film x-ray. DUS at 6 months	DUS<br><br>CTA if sac size increase
16	Pre-discharge: biplanar plain film x-ray. CTA at 6 weeks, CTA at 12 months + biplanar plain film x-ray.	DUS<br><br>CTA if new endoleak and/or sac size increase

	Abnormalities first CTA:<br>○ Type I endoleak: intervention<br>○ Type II endoleak: strict follow-up protocol<br>○ After 6 months: biplanar plain film x-ray + CT, if sac size increase >10% → CTA<br>○ After 12 months: biplanar plain film x-ray + CT<br><br>- No abnormalities first CTA:<br>○ Minimal follow-up<br>○ After 12 months: biplanar plain film x-ray + CT	
