# Supplementary material for: Analysis of Outcomes After Endovascular Abdominal Aortic Aneurysm Repair in Patients With Abnormal Findings on the First Postoperative Computed Tomography Angiography
Source: J Endovasc Ther. 2021 Jul 28;28(6):878–87. doi: 10.1177/15266028211030539 (PMC8573614; doi:10.1177/15266028211030539)
Supplement: sj-pdf-3-jet-10.1177_15266028211030539 – Supplemental material for Analysis of Outcomes After Endovascular Abdominal Aortic Aneurysm Repair in Patients With Abnormal Findings on the First Postoperative Computed Tomography Angiography [file sj-pdf-3-jet-10.1177_15266028211030539.pdf]

Supplemental Table 2: univariable and multivariable analysis after imputation (all-cause mortality and secondary interventions)

	**Univariable analysis**				**Multivariable analysis**			
	Mortality		Secondary interventions		Mortality		Secondary interventions	
	Hazard ratio	*95% CI*	*Hazard ratio*	*95% CI*	*Hazard ratio*	*95% CI*	*Hazard ratio*	*95% CI*
Age*	1.060	1.041-1.079	0.987	0.968-1.007	1.064	1.045-1.083		
Gender	0.956	0.690-1.322	0.783	0.522-1.175				
ASA I/II	Reference	Reference	Reference	Reference	Reference	Reference	Reference	Reference
ASA III	1.070	0.836-1.371	1.187	0.859-1.641	1.100	0.854-1.416	0.945	0.644-1.387
ASA IV*/**	2.899	1.814-4.633	3.166	1.765-5.678	3.203	1.994-5.145	2.598	1.297-5.204
Neck length**	1.034	0.929-1.151	0.792	0.675-0.930			0.790	0.665-0.939
Endurant (Medtronic)	Reference	Reference	Reference	Reference	Reference	Reference	Reference	Reference
Talent (Medtronic)	0.640	0.382-1.072	0.642	0.306-1.346	0.736	0.438-1.235	0.570	0.225-1.444
Excluder (Gore)*/**	0.706	0.505-0.986	0.733	0.467-1.150	0.706	0.505-0.987	0.566	0.327-0.982
Zenith (Cook)*	0.707	0.537-0.930	0.959	0.674-1.365	0.679	0.513-0.898	1.008	0.663-1.532
Other	0.413	0.181-0.939	0.953	0.435-2.087	0.480	0.210-1.093	0.985	0.390-2.486
AAA diameter	1.048	0.949-1.159	1.111	0.979-1.262				

Maximum iliac diameter	1.056	0.894-1.246	1.212	0.993-1.479				
Change in AAA diameter								
Stable or decrease	Reference	Reference	Reference	Reference			Reference	Reference
Increase**	1.013	0.756-1.357	2.335	1.687-3.232			2.429	1.683-3.505

Abbreviations: AAA; abdominal aortic aneurysm, ASA; American Society of Anesthesiologists, CI; confidence interval

*variables significantly related to mortality in multivariable analysis

**variables significantly related to secondary interventions in multivariable analysis
